# The effect of subliminal priming on team trust: The mediating role of perceived trustworthiness

**DOI:** 10.3389/fpsyg.2023.1099267

**Published:** 2023-02-24

**Authors:** Jie Cai, Rongxiu Wu, Jingyu Zhang, Xianghong Sun

**Affiliations:** ^1^CAS Key Laboratory of Behavioral Science, Institute of Psychology, Beijing, China; ^2^Department of Psychology, University of Chinese Academy of Sciences, Beijing, China; ^3^Department of Psychology, Zhejiang Sci-Tech University, Hangzhou, China; ^4^Science Education Department, Harvard-Smithsonian Center for Astrophysics, Harvard University, Cambridge, MA, United States

**Keywords:** mediating effect, public good game, subliminal priming, team trust, perceived trustworthiness

## Abstract

The present study aimed to explore the effect of subliminal priming on team trust and the mechanism through the mediating role of perceived trustworthiness. A total of 144 participants were asked to complete a lexical decision task that was embedded with the “trust” or “suspicion” Chinese words as the subliminal stimuli. Then, they played a public good game and evaluated the perceived trustworthiness of the team. The results of the study showed that subliminal stimuli had a significant effect on team trust [β = –0.99, 95% CI = (−1.64, −0.33)]. Perceived trustworthiness was found to have a significant mediating effect between the priming condition and team trust [β = −0.35, 95% CI = (−0.72, −0.02)]. The current study revealed the underlying mechanism through which subliminal priming techniques influence team trust and informed efforts by altering perceived trustworthiness.

## Introduction

With the speedy development of the world economy and technology, cooperation between countries and organizations around the world has strengthened. Online teams play an increasingly important role in the cooperation between countries and organizations (Gillam and Oppenheim, 2006; Horwitz et al., 2006). Previous studies showed that team trust plays a key role in organizational cooperation and has attracted the attention of more and more researchers (Guzzo and Dickson, 1996; De Jong et al., 2016). Team trust, defined as the positive expectations of team members and willingness to accept vulnerability in the team (Fulmer and Gelfand, 2012), is considered to be the glue of the collective entity with diversified individuals to finish the highly interdependent tasks (Ezz, 2015). A large body of studies suggested that team trust is key to team satisfaction (Costa, 2003), information sharing (Howorth et al., 2004), and team performance (Porter and Lilly, 1996; Webber, 2008; Schaubroeck et al., 2011; Lusher et al., 2014).

Given the importance of team trust, it is of great research interest and necessity to learn more about its predictive factors. For example, trust propensity has been demonstrated to be a crucial factor in determining team trust in newly formed project teams (Costa et al., 2009). Team leaders play a primary role in establishing and developing trust in teams (Dirks and Ferrin, 2002). Organizational climate has also been found to exert a meaningful effect on team trust (Ostroff et al., 2003). Moreover, Barczak et al. (2010) found that “team emotional intelligence promotes team trust. Trust, in turn, fosters a collaborative culture which enhances the creativity of the team.” Current research also showed that team trust could be affected by many aspects of culture, such as language, norms, and so on (Cheng et al., 2016). These factors have been considered explicit factors and well-acknowledged by many researchers.

Except for those explicit factors that have been given much attention in research, to the best of our knowledge, team trust is also greatly affected by some subtle factors such as priming. For example, Ahmed and Hammarstedt (2011) used the scrambled sentence task to prime participants with religious/non-religious words before they were asked to make a one-shot/three-person public goods game that was used to measure team trust and found that priming of religious words increased the level of team trust in the experiment. Drouvelis et al. (2015) used a word-search puzzle task to prime the participants, in which the words were either related to cooperation or not, and they found that, after being primed with cooperative words, the participants would contribute significantly more tokens to the subsequent one-shot/three-person public goods game. Bartke et al. (2019) primed motives of care and anger by way of an autobiographical recall task, and they found that care elicits significantly higher contributions than anger in the public good game. One important caveat with the priming examples aforementioned is that, although the purpose of the research was not revealed, the participants were fully aware of the meaning of the prime words. In other words, these studies mainly focused on the prime words that were all above consciousness; however, whether such an effect can be induced by a purely subliminal process is not known.

Previous studies suggested that subliminal priming can avoid the individual's aversion to supraliminal priming and become an impetus to subsequent actions, therefore changing individuals' behaviors unconsciously (Smeesters et al., 2009). With this in mind, rich theoretical and empirical pieces of evidence on subliminal priming have been accumulated (Collins and Loftus, 1975; Cohen et al., 1988). Moreover, research has found that subliminal priming can affect interpersonal trust (Huang and Murnighan, 2010; Posten et al., 2014; Cai et al., 2020). Previous research showed that “interpersonal trust is directed toward a specific target and team trust refers to trust in a collectivity of interdependent people pursuing a shared goal with inherently unique dynamics.” However, team trust comprises some similar underlying dimensions to interpersonal trust, that is, positive expectation and the willingness to be vulnerable (Rousseau et al., 1998; Fulmer and Gelfand, 2012). Accordingly, we speculated that subliminal priming can also affect team trust.

Furthermore, we addressed the underlying mechanisms of the relationship between subliminal priming and team trust, integrating the finding of prime-to-behavior effects (Wheeler and Demarree, 2009; Wheeler et al., 2011). Prime-to-behavior effects suggest that perception is critical to establish the validity of prime-to-behavior effects (Smeesters et al., 2009). For example, Wheeler and Petty (2001) stated that, when people were present in the priming situation, primes could affect their behavior through the effect on a person's perceptions. Smeesters et al. (2009) concluded that priming effects on behavior in interpersonal contexts were mediated by interpersonal perception. Additionally, Cai et al. (2020) found that the perception of the other partner played a mediating role between the prime and interpersonal trust behavior as measured in the donations in the trust game. Following the view that team perception is shaped by the interaction of individuals in the team (Pype et al., 2018), and combined with the evidence that team trust and interpersonal trust share the same conceptual structure (Rousseau et al., 1998; Fulmer and Gelfand, 2012), we speculated that team perception (Mayer et al., 1995) can also mediate subliminal priming and team trust.

In summary, we adopted a new perspective on the development of team trust in this study, that is, we explored team trust from a subliminal perspective, which is different from previous relevant research on team trust (De Jong and Elfring, 2010; De Jong et al., 2016). It lays a theoretical basis for improving team trust from the subliminal perspective. In addition, this study explored the mechanism of the priming effect and established a theoretical model of subliminal stimuli affecting team trust. Taken together, the current study aimed to (a) explore whether subliminal priming can affect team trust and (b) investigate whether team perception, as measured by perceived trustworthiness to the team, mediates the association between subliminal priming and team trust.

## Methods

### Participants

As suggested by Schoemann et al. (2017), we adopted Monte Carlo power analysis to calculate the sample size under the condition of one mediator and power with 0.5, and at least 127 participants should be included. As such, a total of 144 participants were recruited to complete this experiment from the University of Chinese Academy of Sciences and Zhejiang Sci-Tech University with an advertisement for recruiting participants through posters on campuses. Before the experiment, we obtained consent from all participants, in which they stated that they participated in the experiment voluntarily and they had never participated in similar experiments before. A total of 136 participants (61 men, 75 women) whose ages ranged from 16 to 35 years (*M* = 20.17, *SD* = 2.83) were taken as the final study sample since there were eight participants who did not pass the awareness check. The whole experiment lasted 20 to 30 min, and every participant received ¥20 to ¥30 as a reward, depending on the actual completion time of the specific experiment. The recruitment procedure and research protocol were approved by the Institutional Review Board of the Institute of Psychology, Chinese Academy of Sciences.

### Design

A single-factor design was applied in this experiment. The independent variable was the priming condition, which had two levels: trust priming and suspicion priming. The dependent variable was team trust, which was measured by the number of tokens invested in the public account (Evans and Krueger, 2009). The mediator variable was perceived trustworthiness. The control variable was trust propensity.

### Materials

Participants were seated in individual cubicles in front of personal computers equipped with 60-Hertz/1280 × 1024- pixel monitors, and the distance from participants' eyes to the center of the screen was approximately 60 cm. All materials were presented with E-prime 2.0 (Psychology Software Tools, Inc.).

### Procedure

#### Prime task

**Figure 1:**
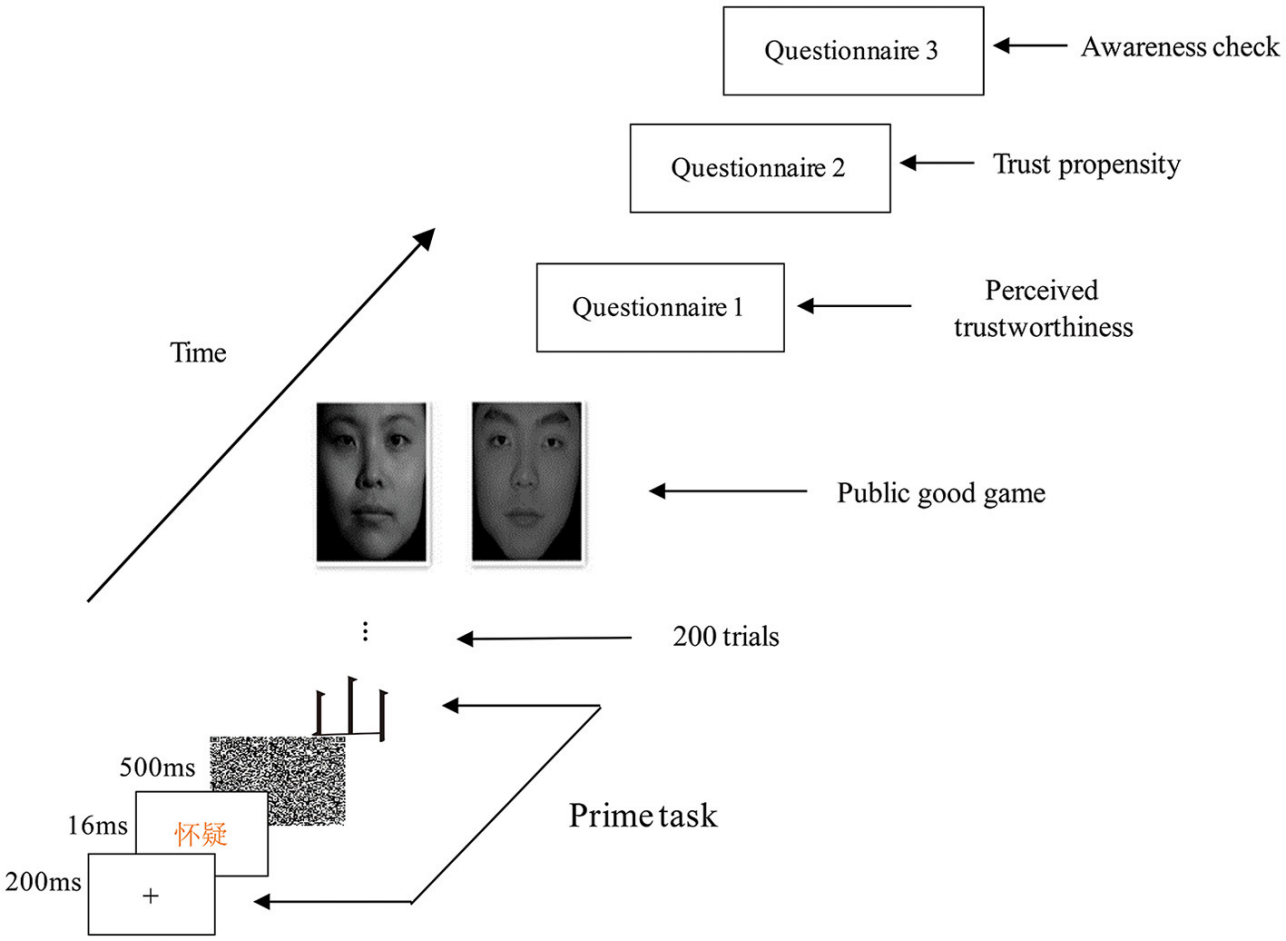
Illustration of the whole process.

Participants were first asked to perform a lexical decision task, in which they had to distinguish whether the presented strings were Chinese characters. The subliminal priming was embedded in this lexical decision task. At the beginning of each trial, a fixation marker “+” appeared in the center of the screen and lasted for 200 ms. The priming words would show on the screen for 16 ms. For the trust priming condition, the word was the Chinese word “信任 ” [trust]. For the suspicion priming condition, it was “怀疑 ” [distrust]. Then, a post-mask string would show for 500 ms. Finally, the target string appeared on the screen until the participants made their lexical choice. Participants were required to press “z” when they saw Chinese characters and “m” when they saw other kinds of strings (Japanese katakana). There were 200 trials, including 150 trials with Chinese words and another 50 trials with non-Chinese words. All these targeted words were not related to the meaning of trust. Figure 1 illustrates the whole process.

#### The public good game

After completing the priming task, the participants would play a single-shot public good game with a virtual team member. There was a public (team) account. In the beginning, all of the team members started with 8 endowed tokens. Next, all the team members could invest a certain number of tokens into the public account separately at the same time. The number of all tokens sent to the public account would become two times larger and were divided between each team member. For example, if a participant invested 4 tokens and the other two team members invested 8 and 6 tokens, the public account would receive (4 + 8 + 6) ^*^2 = 36 tokens, and each player would get 12 tokens back. Then, participants would receive 8 more tokens than in their initial condition (8 – 4 + 12 = 16 tokens), which captured the value of trusting his/her team members. However, if a participant invested 4 tokens and the other two team members did not invest in any token, then the public account would receive only (4 + 0 + 0) ^*^ 2 = 8 tokens, and each team member would receive a return of 2.6 tokens. In this situation, the participant would have only 8 – 4 + 2.6 = 6.6 tokens, while the other two team members would have 10.6 tokens. Participants were told that, if they received more tokens, they would receive a higher payment later (though not knowing the actual exchange rate). Finally, all participants were told how many tokens they would receive (the quantity was assigned randomly). By playing the game, participants can see a photograph of each player. The photographs (one man and one woman with neutral expressions) were taken from the Chinese Affective Picture System (Bai et al., 2005).

#### Perceived trustworthiness

Perceived trustworthiness was measured by one item that was adapted from the measurement proposed by Mayer et al. (1995). The item was “How trustworthy do you think the other team members are,” with 1 representing *the lowest trustworthiness* and 9 representing *the highest trustworthiness*. Each participant was asked to choose one option between 1 and 9 based on their feeling.

#### Trust propensity

Trust propensity is a stable trait that the trustor exhibits toward the trustee, which can influence the trust process (McKnight et al., 1998). In this study, we took trust propensity as a control variable to ensure the reliability of the experimental results. As such, the Chinese version of the interpersonal trust scale was assessed after the measurement of perceived trustworthiness (Rotter, 1967; Wang et al., 1999).

#### Awareness check

We followed up by checking with the participants if they were aware of the priming word using the method proposed by Posten et al. (2014). They were asked to read a 10-word list containing the prime word and to point out which word in the list had been presented to them.

### Data analysis

First, we conducted a covariance analysis to evaluate the main effect of the priming condition. Second, we used Pearson's correlation analysis to test the relationships among the measured variables. Finally, we assessed the mediating effects of perceived trustworthiness following the recommendation of Hayes (2017).

## Results

### Effect of priming on team trust

The results showed that participants in the trust prime condition (*M* = 4.70, *SD* = 2.00) invested more tokens into the public (team) account than those for the participants in the suspicion prime condition (*M* = 3.72, *SD* = 1.76), *F* (1) = 8.96, *p* =.003, η^2^_*p*_ =.06.

### Correlation analysis

Taking trust propensity as a control variable, the partial correlation was adopted to investigate the relationship among priming condition, perceived trustworthiness, and team trust. As shown in Table 1, the priming condition had a statistically significant correlation with perceived trustworthiness (*r* = −0.18, *p* < 0.05) and team trust (*r* = −0.25, *p* < 0.01). Additionally, team trust had a statistically significant correlation with perceived trustworthiness (*r* = 0.54, *p* < 0.001).

**Table 1:** Means, standard deviations, and correlations (*N* = 136).

	***M***	**SD**	**1**	**2**	**3**
1. Priming condition	1.51	0.50	-		
2. Perceived trustworthiness	5.06	1.66	−0.18^*^	-	
3. Team trust	4.21	1.94	0.25^**^	0.54^***^	-

^*^p < 0.05, ^**^p < 0.01, ^***^p < 0.001.

### Mediation analysis

**Figure 2:**
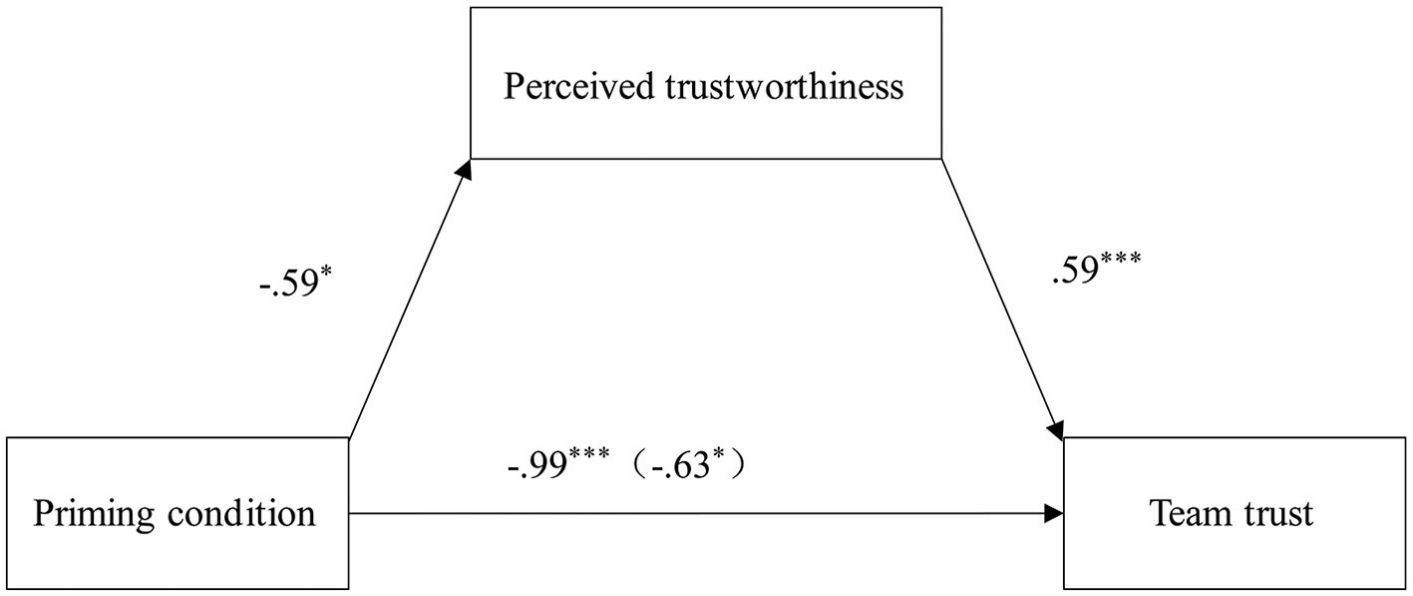
Illustration of the mediation effect. *p < 0.05, ***p < 0.001.

Adopting Hayes (2017) method, a mediation analysis (Model 4 in Process macro) with a bootstrapping method (5,000 samples) was performed. The total effect of the priming condition on team trust was β = −0.99 [95% CI = (−1.64, −0.33)]. However, when the mediator was added to the analysis, the direct effect of the priming condition dropped to β = −0.63 [95% CI = (−1.19, −0.59)]. Meanwhile, the priming condition had a statistically significant effect on perceived trustworthiness [β = −0.59, 95% CI = (−1.16, −0.03)], perceived trustworthiness also had a significant effect on team trust [β = 0.59, 95% CI = (0.43, 0.77)], and perceived trustworthiness was found to have a significant mediating effect between priming condition and team trust [β = −0.35, 95% CI = (−0.72, −0.02)]. The relationship is depicted in Figure 2.

## Discussion

The current study sought to explore the effect of subliminal priming on team trust and whether perceived trustworthiness could mediate this effect. The participants were primed with either a trust or a non-trust word, after which they performed a public good game and perceived trustworthiness was measured. We found evidence to support the effect of subliminal priming on team trust and the mediating role of perceived trustworthiness.

The results showed that subliminal priming had a significant impact on team trust. Specifically, after the concept of trust (vs. suspicion) was subliminally primed, participants invested more (vs. less) tokens in the public account. It was consistent with previous results regarding the impact of subliminal priming on interpersonal trust (Huang and Murnighan, 2010; Posten et al., 2014; Cai et al., 2020), which possibly suggested that the effect was consistent across different types of trust. This finding was also consistent with the idea that cognitive content can be unconsciously activated and guide behavior outside of conscious awareness (Rusbult and Van Lange, 2003). The authors indicated that the effect existed at the behavioral level; in other words, team trust behavior could be given impetus by unconscious stimuli, of which the individual themselves was not aware. Furthermore, this finding was possibly related to the predictive process of the brain (Bubic et al., 2010). Predictive processing is a brain activation triggered by a specific task cue that precedes the presentation of target stimuli (Simmons et al., 2004). This activation is generated by learning the presentation of stimuli related to target stimuli in a short period of time (Schubotz and Von Cramon, 2001). The predictive processing forms a contextual structural relationship with the target stimuli and provides expectations about the stimuli attribute, thus promoting perception and behavior (Bar, 2004). In other words, subliminal stimuli form a contextual structural relationship with the subsequent trust behavior in this study, providing an expectation for subsequent trust behavior and then making an impact on trust behavior. Moreover, in the mindsponge theory, it states that “an information particle must exist within a mind (the subjective world) to be processed by the mind” and “mind is analogized to a sponge that absorbs new compatible values and squeezes out incompatible values with its core values” (Vuong and Napier, 2015; Le et al., 2022; Nguyen et al., 2022). The innermost part of mind represents a set of core values, which can make a great impact on the perceptions, attitudes, and behaviors of each individual (Nguyen et al., 2022). The information processing mechanism within the mind filters the information to enter the innermost part of the mind based on the gatekeeper role of trust. New information that has been trusted will have either a priority to pass into the innermost part of the mind or a priority to refuse (Nguyen et al., 2022). In this study, we manipulated subliminal information, and other factors such as consciousness information were controlled. That is to say, the participants cannot conduct trust evaluations based on the information above consciousness. Subliminal information has become the only source of information that can be used for trust evaluation. Specifically, when conducting trust evaluation, individuals absorb the subliminal information in the environment and examine the compatibility with their own core values. It should be noted that conscious awareness is not necessary in this process. In this study, the Chinese words presented subliminally become the reference basis for the participants to conduct the trust evaluation. Therefore, accordingly, the study result also abides by the mindsponge theory's view. Overall, we demonstrated that primed with trust (vs. suspicion) subliminally can shape subsequent team trust behavior within the Chinese sample using the Chinese language, providing an experimental basis for improving team trust.

Another finding obtained from this study was that subliminal priming had a significant impact on perceived trustworthiness. This finding was consistent with previous results (Todorov et al., 2009; Posten et al., 2014), which indicated that trust judgments can be modified without the conscious process but modified through the activation of cognitive concepts at the unconscious level. Moreover, perceived trustworthiness showed a positive relationship with trust. Perceived trustworthiness is found to be the most important factor in predicting an individual's trust behavior, which is consistent with that mentioned in previous studies (Mayer et al., 1995; Robbins, 2016). Therefore, this study verified one more time that perceived trustworthiness is an important factor for prompting changes in team trust behavior.

The last finding from our research suggested that perceived trustworthiness played a mediating role between subliminal priming and team trust. The prime-to-behavior effects hold that priming affects behavior on the premise that priming can affect perception, which, in turn, affects behavior (Wheeler and Demarree, 2009). For example, the individual primed with friendly related concepts could activate perceptions of the other person, and then, the individual shows more friendly behavior in subsequent tasks (Smeesters et al., 2009). This finding suggests that the unconscious influence may emerge to a conscious level, and may influence people's perception, which, in turn, changes their subsequent behaviors. In other words, the individual primed with subliminal stimuli might take this unconscious information as a cue to shape the perceived trustworthiness of other individuals and then make trust behavior based on the perceived trustworthiness. It indicated that perceived trustworthiness may be one of the reasons why subliminal stimuli affect team trust.

To conclude, this study made contributions to the literature from two aspects. First, this research theoretically supplemented the predictive sources of team trust and extended the extant theory (e.g., mindsponge theory) on the subliminal priming of team trust and may provide a novel way to adjust team trust, especially for virtual teams that needed to communicate online in the current COVID-19 context. Second, although many studies showed that perceived trustworthiness is an important source of trust behavior (Mayer et al., 1995), this research initially integrated the finding of prime-to-behavior effects, established a theoretical model among subliminal priming, perceived trustworthiness, and team trust, and finally revealed the internal mechanism of subliminal priming on team trust.

### Limitations and future research directions

The present study revealed some interesting findings; however, the limitations of this study are still worth discussing. First, the participants were all young adults aged 16–35 years. Previous studies suggested that trust changes with age (Belli et al., 2012). Future studies are encouraged to explore the universality of this effect by expanding the investigation to other age groups. Second, we followed the procedure of a previous study by not setting a control group (Posten et al., 2014). As a result, it is not clear whether there is a difference between the two priming conditions that resulted from an enhancing effect or an undermining effect. However, we cannot deny that the current research findings represented an important initial step for exploratory research to find out the difference between strong conditions. Future studies are needed to set up a control group to deepen the understanding of this effect. Third, although we found that unconscious priming could affect people's conscious perception and then lead to behavioral changes, we could not exclude the possibility that unconscious priming affects behavior *via* unconscious perception. Perhaps, we can change the perception measurement to explore whether unconscious priming can affect behavior through unconscious perception in the future. Finally, trust propensity was used as a control variable to eliminate the confusion effect in this study. However, previous studies showed that culture has a significant impact on team trust. Specifically, compared with Western culture, trust behavior and trustworthiness perception would be higher in the context of Chinese culture (Buchan et al., 2002; Kiyonari and Cook, 2006). Therefore, it is necessary to take cultural factors related to team trust into account to expand the universality of this effect in different cultures in future research.

## Conclusion

This study attempted to explore the relationship between subliminal priming, perceived trustworthiness, and team trust. We demonstrated that subliminal priming had a significant impact on team trust, and perceived trustworthiness played a mediating role in the effect of subliminal priming on team trust. It represents one of the hosts of novel directions in team trust and illustrates the potential ubiquity of subliminal information in affecting team trust.

## Data availability statement

The raw data supporting the conclusions of this article will be made available by the authors, without undue reservation.

## Ethics statement

The study was approved by the Human Research Ethics Committee of Zhejiang Sci-Tech University. The patients/participants provided their written informed consent to participate in this study. Written informed consent was obtained from the individual(s) for the publication of any potentially identifiable images or data included in this article.

## Author contributions

Contributed to conception and design: JC, JZ, and XS. Contributed to the acquisition of data: JC. Contributed to analysis and interpretation of data: JC and RW. Drafted and/or revised the article: JZ, XS, and RW. Approved the submitted version for publication: JC, RW, JZ, and XS.

## References

[B1] Ahmed A. Hammarstedt M. (2011). The effect of subtle religious representations on cooperation. Int. J. Soc. Econ. 38, 900–910. 10.1108/03068291111171405

[B2] Bai L. Ma H. Huang Y. Luo Y. (2005). The development of Native Chinese Affective Picture System—a pretest in 46 college students. Chin. Ment. Health J. 19, 719–722. 10.00-6729/(2005)11-719-04

[B3] Bar M. (2004). Visual objects in context. Nat. Rev. Neurosci. 5, 617–629. 10.1038/nrn1476 15263892

[B4] Barczak G. Lassk F. Mulki J. (2010). Antecedents of team creativity: An examination of team emotional intelligence, team trust and collaborative culture. Creat. Innov. Manag. 19, 332–345. 10.1111/j.1467-8691.2010.00574.x

[B5] Bartke S. Bosworth S. J. Snower D. J. Chierchia G. (2019). Motives and comprehension in a public goods game with induced emotions. Theory Dec. 86, 205–238. 10.1007/s11238-018-9677-5

[B6] Belli S. R. Rogers R. D. Lau J. Y. F. (2012). Adult and adolescent social reciprocity: experimental data from the Trust Game. J. Adol. 35, 1341–1349. 10.1016/j.adolescence.2012.05.004 22691532

[B7] Bubic A. Yves von Cramon D. Schubotz R. I. (2010). Prediction, cognition and the brain. Front. Hum. Neurosci. 4, 1–15. 10.3389/fnhum.2010.00025 20631856 PMC2904053

[B8] Buchan N. R. Croson R. T. A. Dawes R. M. Buchan N. R. (2002). Swift neighbors and persistent strangers: a cross-cultural investigation of trust and reciprocity in social exchange. Am. J. Sociol. 108, 168–206. 10.1086/344546

[B9] Cai J. Zhang J. Sun X. (2020). Influence of subliminal stimuli on interpersonal trust: a possible mechanism. PsyCh J. 9, 644–650. 10.1002/pchj.364 32830453

[B10] Cheng X. Fu S. Sun J. Han Y. Shen J. Zarifis A. (2016). Investigating individual trust in semi-virtual collaboration of multicultural and unicultural teams. Comp. Hum. Behav. 62, 267–276. 10.1016/j.chb.2016.03.093

[B11] Cohen J. Dunbar K. Mcclelland J. (1988). On the control of automatic processes: a parallel distributed processing model of the Stroop effect. Psychol. Rev. 97, 332–361. 10.1037/0033-295X.97.3.332 2200075

[B12] Collins A. M. Loftus E. F. (1975). A spreading-activation theory of semantic processing. Psychol. Rev. 82, 407–428. 10.1037/0033-295X.82.6.407

[B13] Costa A. C. (2003). Work team trust and effectiveness. Person. Rev. 32, 605–622. 10.1108/00483480310488360

[B14] Costa A. C. Bijlsma-Frankema K. M. de Jong B. A. (2009). The role of social capital on trust development and dynamics: implications for cooperation, monitoring and team performance. Soc. Sci. Inform. 48, 199–228. 10.1177/0539018409102408

[B15] De Jong B. Elfring T. (2010). How does trust affect the performance of ongoing teams? The mediating role of reflexivity, monitoring and effort. Acad. Manag. J. 53, 535–549. 10.5465/AMJ.2010.51468649

[B16] De Jong B. A. Dirks K. T. Gillespie N. (2016). Trust and team performance: A meta-analysis of main effects, moderators, and covariates. J. Appl. Psychol. 101, 1134. 10.1037/apl0000110 27123697

[B17] Dirks K. T. Ferrin D. L. (2002). Trust in leadership: meta-analytic findings and implications for research and practice. J. Appl. Psychol. 87, 611–628. 10.1037/0021-9010.87.4.611 12184567

[B18] Drouvelis M. Metcalfe R. Powdthavee N. (2015). Can priming cooperation increase public good contributions? Theory Dec. 79, 479–492. 10.1007/s11238-015-9481-4

[B19] Evans A. M. Krueger J. I. (2009). The psychology (and economics) of trust. Soc. Person. Psychol. Compass 6, 1003–1017. 10.1111/j.1751-9004.2009.00232.x

[B20] Ezz M. E. (2015). Global Virtual Teams: Trust and Communication as Facilitators and Barriers to Performance and Team Conflict Resolution. College Park, MD: University of Maryland University College.

[B21] Fulmer C. A. Gelfand M. J. (2012). At what level (and in whom) we trust: trust across multiple organizational levels. J. Manag. 38, 1167–1230. 10.1177/014920631243932732884473

[B22] Gillam C. Oppenheim C. (2006). Review article: Reviewing the impact of virtual teams in the information age. J. Inform. Sci. 32, 160–175. 10.1177/0165551506062328

[B23] Guzzo R. A. Dickson M. W. (1996). Teams in organizations: recent research on performance and effectiveness. Annu. Rev. Psychol. 47, 307–338. 10.1146/annurev.psych.47.1.307 15012484

[B24] Hayes A. F. (2017). Introduction to Mediation, Moderation, and Conditional Process Analysis: A Regression-Based Approach. New York, NY: Guilford Press.

[B25] Horwitz F. M. Bravington D. Silvis U. (2006). The promise of virtual teams: Identifying key factors in effectiveness and failure. J. Eur. Indust. Train. 30, 472–494. 10.1108/03090590610688843

[B26] Howorth C. Westhead P. Wright M. (2004). Buyouts, information asymmetry and the family management dyad. J. Busin. Vent. 19, 509–534. 10.1016/j.jbusvent.2003.04.002

[B27] Huang L. Murnighan J. K. (2010). What's in a name? Subliminally activating trusting behavior. Organ. Behav. Hum. Decis. Process. 111, 62–70. 10.1016/j.obhdp.2009.10.002

[B28] Kiyonari T. Cook K. S. (2006). Does Trust Beget Trustworthiness? Trust and trustworthiness in two games and two cultures : a research note. Soc. Psychol. Q. 69, 270–283. 10.1177/019027250606900304

[B29] Le T.-T. Nguyen M.-H. Vuong Q.-H. (2022). Chapter 4: trust in mindsponge: a new perspective on information reliability. In: Vuong, Q.-H., La, V.-P., and Nguyen, M.-H. The Mindsponge and BMF Analytics for Innovative Thinking in Social Sciences and Humanities. Berlin: De Gruyter. p. 67–86.

[B30] Lusher D. Kremer P. Robins G. (2014). Cooperative and competitive structures of trust relations in teams. Small Group Res. 45, 3–36. 10.1177/1046496413510362

[B31] Mayer R. C. Davis J. H. Schoorman F. D. (1995). An integration model of organizational trust. Acad. Manag. Rev. 20, 709–734. 10.2307/258792

[B32] McKnight D. H. Cummings L. Chervany N. L. (1998). Initial trust formation in new organizational relationships. Acad. Manag. Rev. 23, 473. 10.2307/259290

[B33] Nguyen M. H. La V. P. Le T. T. Vuong Q. H. (2022). Introduction to Bayesian mindsponge framework analytics: an innovative method for social and psychological research. MethodsX 9, 101808. 10.1016/j.mex.2022.101808 36034522 PMC9400117

[B34] Ostroff C. Kinicki A. J. Tamkins M. M. (2003). Organizational Culture and Climate. London: Wiley.

[B35] Porter T. W. Lilly B. S. (1996). The effects of conflict, trust, and task commitment on project team performance. Int. J. Conflict Manag. 7, 361–376. 10.1108/eb022787

[B36] Posten A.-C. Ockenfels A. Mussweiler T. (2014). How activating cognitive content shapes trust: a subliminal priming study. J. Econ. Psychol. 41, 12–19. 10.1016/j.joep.2013.04.002

[B37] Pype P. Mertens F. Helewaut F. Krystallidou D. (2018). Healthcare teams as complex adaptive systems: Understanding team behaviour through team members' perception of interpersonal interaction. BMC Health Serv. Res. 18, 1–13. 10.1186/s12913-018-3392-3 30029638 PMC6053823

[B38] Robbins B. G. (2016). Probing the links between trustworthiness, trust, and emotion : evidence from four survey experiments. Soc. Psychol. Q. 79, 284–308. 10.1177/0190272516657546

[B39] Rotter J. B. (1967). A new scale for the measurement of interpersonal trust. J. Pers. 35, 651–665. 10.1111/j.1467-6494.1967.tb01454.x 4865583

[B40] Rousseau D. M. Sitkin S. Burt R. S. Camerer C. (1998). Not so different after all: a cross-discipline view of trust. Acad. Manag. Rev. 23, 393–404. 10.5465/amr.1998.926617

[B41] Rusbult C. E. Van Lange P. A. M. (2003). Interdependence, interaction, and relationships. Annual Rev. Psychol. 54, 351–375. 10.1146/annurev.psych.54.101601.145059 12415073

[B42] Schaubroeck J. Lam S. S. Peng A. C. (2011). Cognition-based and affect-based trust as mediators of leader behavior influences on team performance. J. Appl. Psychol. 96, 863–871. 10.1037/a0022625 21299271

[B43] Schoemann A. M. Boulton A. J. Short S. D. (2017). Determining power and sample size for simple and complex mediation models. Soc. Psychol. Person. Sci. 8, 379–386. 10.1177/1948550617715068

[B44] Schubotz R. I. Von Cramon D. Y. (2001). Interval and ordinal properties of sequences are associated with distinct premotor areas. Cereb. Cortex 11, 210–222. 10.1093/cercor/11.3.210 11230093

[B45] Simmons A. Matthews S. C. Stein M. B. Paulus M. P. (2004). Anticipation of emotionally aversive visual stimuli activates right insula. NeuroReport, 15, 2261–2265. 10.1097/00001756-200410050-00024 15371746

[B46] Smeesters D. Wheeler S. C. Kay A. C. (2009). The role of interpersonal perceptions in the prime-to-behavior pathway. J. Person. Soc. Psychol. 96, 395–414. 10.1037/a0012959 19159139

[B47] Todorov A. Pakrashi M. Oosterhof N. N. (2009). Evaluating faces on trustworthiness after minimal time exposure. Soc. Cogn. 27, 813–833. 10.1521/soco.2009.27.6.813

[B48] Vuong Q. H. Napier N. K. (2015). Acculturation and global mindsponge: an emerging market perspective. Int. J. Intercult. Rel. 49, 354–367. 10.1016/j.ijintrel.2015.06.003

[B49] Wang X. D. Wang X. L. Ma H. (1999). Manual of mental health rating scale. Chin. Ment. Health J. 13, 31–35.

[B50] Webber S. S. (2008). Development of cognitive and affective trust in teams: a longitudinal study. Small Group Res. 39, 746–769. 10.1177/104649640832356931178767

[B51] Wheeler S. C. Demarree K. G. (2009). Multiple mechanisms of prime-to-behavior effects. Soc. Person. Psychol. Comp. 3, 566–581. 10.1111/j.1751-9004.2009.00187.x 18453463

[B52] Wheeler S. C. Petty R. E. (2001). The effects of stereotype activation on behavior : a review of possible mechanisms. Psychol. Bull. 127, 797–826. 10.1037/0033-2909.127.6.797 11726072

[B53] Wheeler S. C. Smeesters D. Kay A. C. (2011). Culture modifies the operation of prime-to-behavior effects. J. Exp. Soc. Psychol. 47, 824–829. 10.1016/j.jesp.2011.02.018

